# Heterotopic Ossification of the Peroneus Longus Tendon in the Retromalleolar Portion with the Peroneus Quartus Muscle: A Case Report

**DOI:** 10.1155/2018/7978369

**Published:** 2018-07-29

**Authors:** Song Ho Chang, Takumi Matsumoto, Koichi Okajima, Masashi Naito, Jun Hirose, Sakae Tanaka

**Affiliations:** Department of Orthopaedic Surgery, Faculty of Medicine, The University of Tokyo, 7-3-1 Hongo, Bunkyo-ku, Tokyo 113-8655, Japan

## Abstract

Heterotopic ossification (HO) is an ectopic formation of the lamellar bone in the soft tissues. Some authors have previously reported HO or calcific tendinitis of the peroneus longus tendon at the level of the cuboid bone, while the HO of the peroneus longus tendon in the retromalleolar portion has not been reported. The purpose of this report is to describe clinical, radiological, and histological features of this rare ossification and its treatment. To the best of our knowledge, this is the first report presenting a case of HO of the peroneus longus tendon, which developed in the retromalleolar portion.

## 1. Introduction

Heterotopic ossification (HO) is a pathologic condition in which the ectopic lamellar bone forms within the tendon, muscle, or other soft tissues. The etiology of HO has been classified into three groups: traumatic, neurologic, and genetic [1]. HO commonly involves the ankle joint. HO of the Achilles tendon has been typically reported by some authors [2]. Conversely, HO of the peroneus longus tendon is a relatively rare entity. A previous report referring to ossification or calcification of the peroneus longus tendon has focused and reported on lesions beneath the plantar aspect of the cuboid, lateral to the calcaneus, or at the level of the calcaneocuboid joint [3]. To the best of our knowledge, this is the first report to describe a case of HO of the peroneus longus tendon in the retromalleolar portion successfully resolved through surgical removal.

## 2. Case Presentation

**Figure 1:**
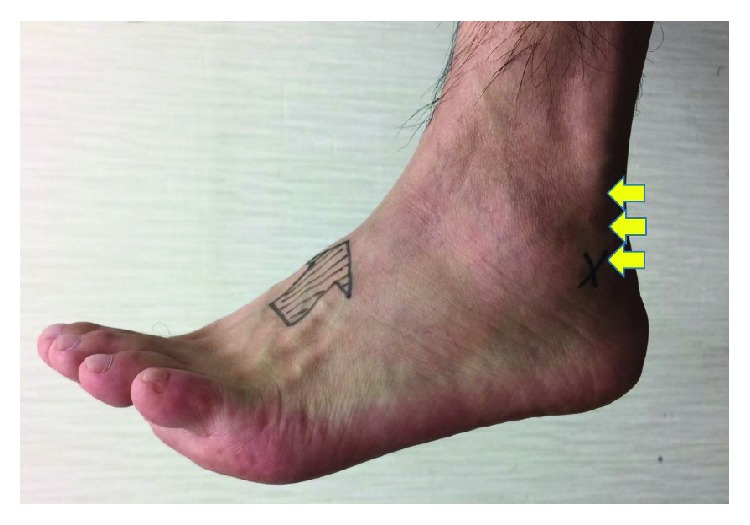
A solid mass 1 × 5 cm in size was palpable over the retromalleolar portion of the left ankle along the course of the peroneal tendons (yellow arrows).

A 50-year-old Japanese man visited a nearby orthopedic clinic complaining of persistent pain during ambulation and solid mass in his lateral retromalleolar portion, which had gradually grown since 5 years prior to visiting our hospital. Conservative treatment, including immobilization using an ankle brace and administration of NSAIDs, failed to reduce his persistent pain, and the patient was then referred to our hospital for surgical treatment. He had a medical history of severe left ankle sprain 35 years prior, which was treated with only bandage application. He was also diagnosed with rheumatoid arthritis 5 years prior at a nearby hospital, which was not treated with antirheumatic drugs. On the first visit to our hospital, his blood test showed the following results: CRP, 0.67 mg/L; RF, 394 IU/mL; MMP-3, 138 ng/mL; and anti-CCP, 363 U/mL. Physical examination revealed a solid mass sized 1 × 5 cm over the retromalleolar portion of the left ankle along the course of the peroneal tendons (Figure 1). He had tenderness and slight swelling on the left retromalleolar space, but no local heat or redness. He had no joint swelling and pain other than the swelling on the left lateral retromalleolar area. Pain was elicited by active plantar flexion of the ankle and eversion of the foot. The range of motion of his left ankle was 5° of dorsiflexion and 35° of plantar flexion, which was limited compared with 10° of dorsiflexion and 45° of plantar flexion of his right ankle with his knees flexed. He had no instability in his ankle joint on the manual anterior drawer test.

**Figure 2:**
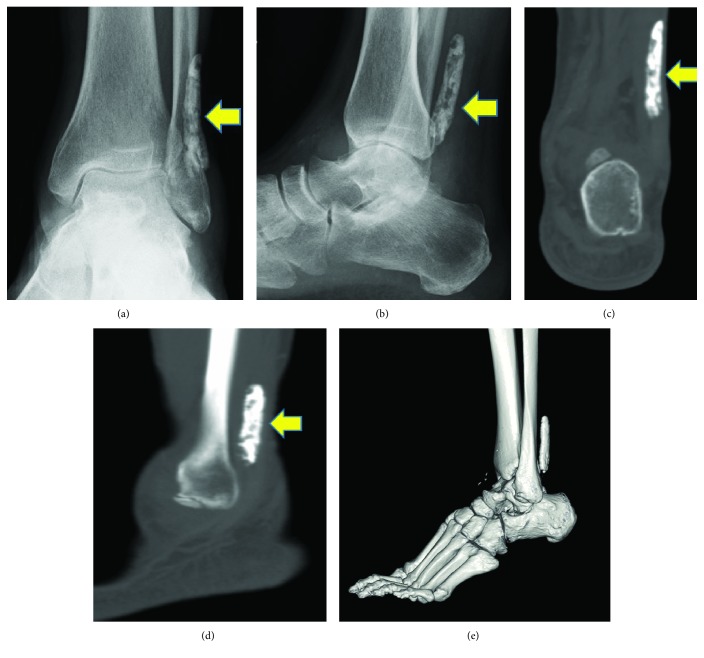
X-ray and CT showed a 1 × 5 cm elliptical opacification (yellow arrow) along the course of the peroneal tendon. (a) AP view of the X-ray image. (b) Lateral view of the X-ray image. (c) Coronal section of the CT image. (d) Axial section of the CT image. (e) 3D reconstruction of the CT image.

**Figure 3:**
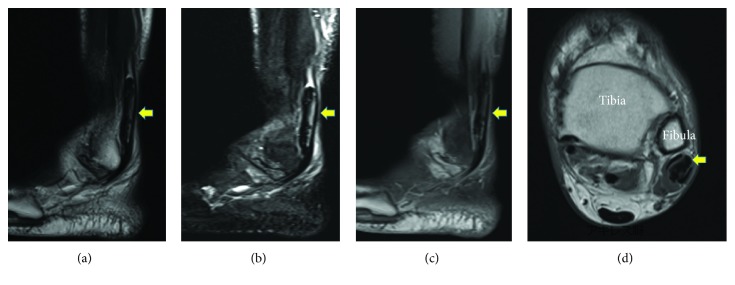
Sagittal T1- and T2-weighted MR images showed an elliptical mass of low intensity partially with high intensity with no contrast effect. Axial T1-weighted MR images showed a low-intensity mass (yellow arrow) in the peroneal tendon sheath, which seemed to compress both the peroneal brevis and longus tendons. (a) Sagittal T1-weighted image. (b) Sagittal T2-weighted image. (c) Sagittal enhanced image. (d) Axial T1-weighted image.

**Figure 4:**
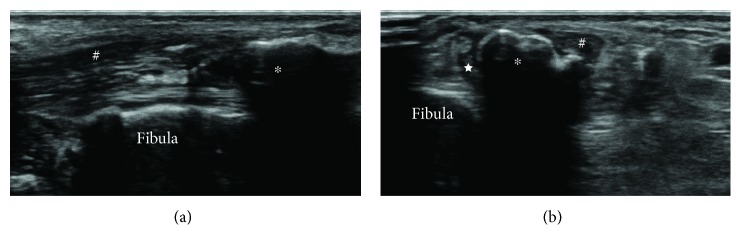
Ultrasonographic images showed an elliptical mass with an echoic shadow on the affected side of the peroneal tendon sheath. (a) Longitudinal image and (b) transverse image. ^∗^Heterotopic ossification. ^#^Peroneus longus tendon. ^★^Peroneus brevis tendon.

X-ray and CT showed a 1 × 5 cm elliptical opacification along the course of the peroneal tendon from the level of the ankle joint at its distal end (Figure 2). Sagittal T1- and T2-weighted MR images showed an elliptical mass of a low intensity partially with high intensity with no contrast effect. Axial T1-weighted MR images showed a low-intensity mass in the peroneal tendon sheath, which seemed to compress both the peroneal brevis and longus tendons (Figure 3). Ultrasonographic image showed an elliptical mass with an echoic shadow on the affected side of the peroneal tendon sheath (Figure 4). We assumed that the mechanism of the present symptom was due to HO or calcinosis in the peroneal tendon sheath. Because of intractable pain and inability to walk, he hoped for a surgical treatment.

**Figure 5:**
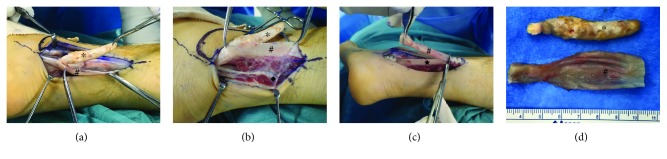
Resection of heterotopic ossification of the peroneus longus and peroneus quartus muscle. (a) Heterotopic ossification existed inside the peroneus longus tendon. (b) The peroneus quartus muscle running along the peroneus longus tendon was excised. (c) An end-to-side transfer of the peroneus longus tendon to the peroneus brevis tendon was performed. The degenerated part of the peroneus tendon was excised. (d) Resected heterotopic ossification and degenerated part of the peroneus longus tendon. ^∗^Heterotopic ossification. ^#^Peroneus longus tendon. ^◆^Peroneus quartus muscle. ^★^Peroneus brevis tendon.

Surgery was performed through the retromalleolar curvilinear approach with a longitudinal 9 cm skin incision. The superior peroneal retinaculum and peroneal tendon sheath were incised. Longitudinal incision of the peroneus longus tendon revealed that the mass existed inside the peroneus longus tendon and its distal end was connected to the peroneus longus tendon (Figure 5). We excised the solid mass and a part of the peroneus longus tendon because no viable tendon remained in this part. The peroneus brevis tendon was intact, without any pathologic features. Because of the large defect after retraction of the peroneus longus tendon, an end-to-side transfer of the peroneus longus tendon to the peroneus brevis tendon was performed distally and proximally using monocryl sutures. We found a peroneus quartus muscle running along the peroneus longus tendon and subsequently excised it (Figure 5). The wound was irrigated and closed, and a sterile dressing was applied.

**Figure 6:**
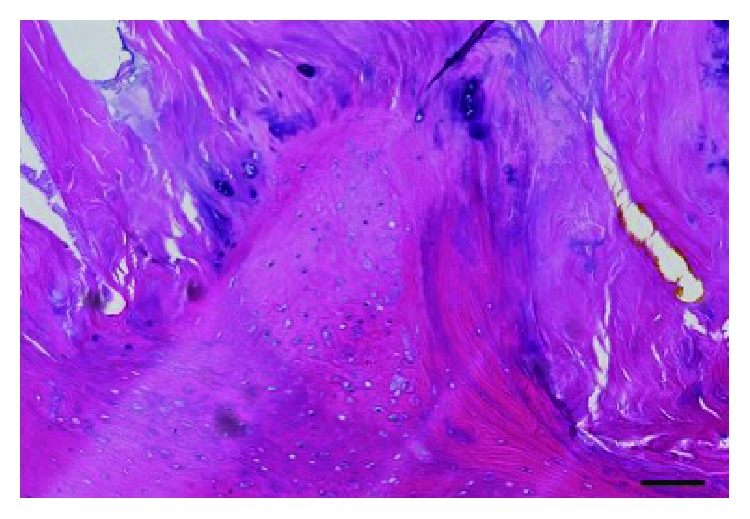
Microphotograph of the resected heterotopic ossification. Lamellar bone formation with an extensive cartilage metaplasia partly with mixed tissue of fat and necrotic muscle. Calcification and cartilage metaplasia existed in the transitional zone between the ossification and the remaining tendon, that is, endochondral ossification. Scale bar, 100 *μ*m.

Microscopic examination revealed a lamellar bone formation with mixed tissue of fat and necrotic muscle. Calcification and cartilage metaplasia existed in the transitional zone between the ossification and the remaining tendon, that is, endochondral ossification (Figure 6). The pathological findings did not indicate intramembranous ossification. The histological diagnosis was HO of the peroneus longus tendon. A below-knee cast was applied on the patient in the operating room; instructions to maintain nonweight bearing for 3 weeks were also provided. After 3 weeks, full-weight bearing was allowed. The cast was removed 6 weeks postoperatively. The postoperative course was uneventful, and the patient returned to normal activities without any kind of functional disability. He started taking antirheumatic drugs at a nearby hospital 2 months after the surgery. We compared the outcomes of the surgery, using an objective standard rating system, the Japanese Society for Surgery of the Foot (JSSF) scale [4, 5], and the Self-Administered Foot Evaluation Questionnaire (SAFE-Q) [6]. The SAFE-Q is an ankle-specific subjective evaluation method consisting of 6 subcategories (i.e., pain and pain-related, physical functioning and daily living, social functioning, shoe-related, general health and well-being, and sports activity [optional]). The preoperative JSSF scale of 54 points (maximum score, 100 points) significantly improved to 100 points after 1 year. Compared to the preoperative condition, all subscale scores in the SAFE-Q improved after 1 year: pain and pain-related, 21 to 100 points; physical functioning and daily living, 50 to 86 points; social functioning, 21 to 88 points; shoe-related 100 to 100 points; and general health and well-being, 25 to 85 points.

## 3. Discussion

HO is defined as the formation of the lamellar bone in the soft tissues, such as the muscle and the joint capsule. Histologic examination of previous reports implicated that HO can be induced by both endochondral ossification and intramembranous ossification [2, 7]. HO occurs twice as frequently in male with no age predilection [8]. It occurs in a variety of conditions, such as previous trauma (tendon rupture or repeated microtrauma) [9], postarthroplasties [10], vasculopathies, central nervous system injury [11], burn injury [12], metabolic conditions, such as diabetes, Wilson's disease and ochronosis, DISH (diffuse idiopathic skeletal hyperostosis), and seronegative arthropathies [13]. In addition, genetic disorders, such as fibrodysplasia ossificans progressiva and progressive osseous heteroplasia, produce multiple HO lesions.

Recent studies have demonstrated that some molecular factors, such as the BMP family, HIF1a, and scleraxis, have a key role in inducing ectopic bone formation [7, 14]; however, several issues remain to be elucidated. It has been suggested that three conditions are required for the formation of HO: [1] presence of an osteoinductive factor, [2] chondrocyte progenitor and osteoblast progenitor cells, and [3] environment permissive to osteogenesis [15]. Under these conditions, mesenchymal cells differentiate into chondrocytes or osteoblasts, which induce HO formation [16].

There is generally no standard treatment for HO. It has been suggested that NSAIDs, localized low-dose irradiation, retinoids, BMP inhibitors, and antagonists have some effect on the prophylaxis of HO formation [7]. These options are effective in preventing HO; however, their efficacy is limited after fibroproliferation and cartilage formation. Surgical resection is the only treatment option to date once bone tissue formation is completed [7].

HO of the peroneal tendon is rare. One report focused on HO of the peroneus brevis tendon in a pediatric patient [17]. Some cases of peroneus longus calcification have been reported [3, 18]; however, all reports revealed calcification of the peroneus longus tendon at the cuboid level. To the best of our knowledge, this is the first report to describe a case of HO of the peroneus longus tendon in the retromalleolar portion.

We found the peroneus quartus muscle running along the peroneus longus tendon in the present case. The peroneus quartus muscle is the most frequent supernumerary muscle around the foot and ankle. A few cases of chronic ankle pain, peroneal tendon sprains, or ankle instability caused by supernumerary muscle were reported. Surgical excision is a favorable treatment option for the symptomatic peroneus quartus muscle [19].

Inflammation following trauma is said to be the first step in the initiation of HO formation [20]. We assume that the triggering factor in the HO formation in the present case could be the severe ankle sprain experienced years prior, and the existence of the peroneus quartus muscle might have helped exacerbate the injury in the peroneus longus tendon. Furthermore, chronic inflammation might be maintained by the uncontrolled rheumatoid arthritis in this case.

In conclusion, we experienced the case of a patient with HO of the peroneus longus tendon in the retromalleolar portion with the peroneus quartus muscle. The present case was successfully treated with surgical removal of HO and the peroneus quartus muscle, together with peroneus longus transfer to the peroneus brevis tendon.
